# Heart Rate Variability and Functional Outcomes of Patients with Spontaneous Intracerebral Hemorrhage

**DOI:** 10.3390/biomedicines12081877

**Published:** 2024-08-16

**Authors:** Kornelia Laichinger, Annerose Mengel, Rebecca Buesink, Sara Roesch, Maria-Ioanna Stefanou, Constanze Single, Till-Karsten Hauser, Markus Krumbholz, Ulf Ziemann, Katharina Feil

**Affiliations:** 1 Department of Neurology & Stroke, Eberhard-Karls University of Tübingen, 72076 Tübingen, Germany; kornelia.laichinger@med.uni-tuebingen.de (K.L.); constanze.single@med.uni-tuebingen.de (C.S.); ulf.ziemann@med.uni-tuebingen.de (U.Z.); katharina.feil@med.uni-tuebingen.de (K.F.); 2 Hertie Institute for Clinical Brain Research, Eberhard-Karls University of Tübingen, 72076 Tübingen, Germany; 3 Department of Neuroradiology, Eberhard-Karls University of Tübingen, 72076 Tübingen, Germany; till-karsten.hauser@med.uni-tuebingen.de; 4 Department of Neurology, University Hospital of the Brandenburg Medical School, 15562 Rüdersdorf, Germany; markus.krumbholz@mhb-fontane.de

**Keywords:** intracerebral hemorrhage, heart rate variability, autonomic dysfunction, functional outcome, big data, intensive care, acute management

## Abstract

Background: The relationship between heart rate variability (HRV) changes potentially indicating autonomic dysregulation following spontaneous intracerebral hemorrhage (ICH) and functional outcome has not yet been fully elucidated. This study investigated the effects of HRV during the initial 96 h after admission on 90-day functional outcome in ICH patients. Methods: We included patients with spontaneous ICH in a prospective cohort single-center study. Continuous HR data were retrieved from the Intellispace Critical Care and Anesthesia information system (Philips Healthcare) and analyzed within the following time intervals: 0–2, 0–8, 0–12, 0–24, 0–48, 0–72, and 8–16, 16–24, 24–48, 48–72, 72–96 h after admission. HRV was determined from all available HR values by calculating the successive variability (SV), standard deviation (SD), and coefficient of variation (CV). Low HRV was set as SD ≤ 11.4 ms, and high HRV as SD > 11.4 ms. The clinical severity of ICH was assessed using the National Institutes of Health Stroke Scale (NIHSS) and functional outcome using the modified Rankin Scale (mRS). Good functional outcome was defined as mRS 0–2. Results: The cohort included 261 ICH patients (mean age ± SD 69.6 ± 16.5 years, 48.7% female, median NIHSS 6 (2, 12), median ICH score 1 (0, 2), of whom 106 (40.6%) had good functional outcome. All patients had the lowest HRV at admission, which increased during the first two days. Comparing ICH patients with low HRV (n = 141) and high HRV (n = 118), those with good outcome showed significantly lower HRV during the first three days (0–72 h: HRV SD good outcome 10.6 ± 3.5 ms vs. poor outcome 12.0 ± 4.0 ms; *p* = 0.004). Logistic regression revealed that advanced age, high premorbid mRS, and high NIHSS at admission were significant predictors of poor functional outcome, while reduced SD of HRV showed a non-significant trend towards good functional outcome (0–72 h: OR 0.898; CI 0.800–1.008; *p* = 0.067). Conclusions: Our results indicate autonomic dysfunction with sympathetic hyperactivity after spontaneous ICH, as reflected by the evidence of the lower HRV in the first days. Initially increased sympathetic tone appears to have a protective effect, as suggested by the comparatively lower HRV in patients with good functional outcome at the first days.

## 1. Introduction

Spontaneous intracerebral hemorrhage (ICH), accounting for 10–15% of all stroke types worldwide, is associated with higher mortality and disability rates compared to acute ischemic stroke [1,2]. The limited therapeutic options for ICH highlight an urgent need for innovative care strategies.

The central autonomic network (CAN) consists of several interconnected areas of the central nervous system (CNS) that control the autonomic output, including the cerebral hemisphere (insular cortex and amygdala), hypothalamus, brainstem (rostral ventrolateral medulla, periaqueductal gray matter, parabrachial complex, and nucleus tractus solitarius), and the lateral horn of the spinal cord [3,4,5]. A lesion in these areas, particularly the cerebral hemispheres with disconnection of the insular cortex, can lead to increased sympathetic output via elimination of tonic inhibition [4,6,7]. The hypothalamus, known as the subcortical center of the autonomic nervous system (ANS), contains the paraventricular nucleus, which regulates sympathetic activity [4,7]. The brainstem contains several autonomic structures: the ventrolateral medulla, an essential regulatory center for heart rhythm and blood pressure; the nucleus tractus solitarii and the rostral ventrolateral medulla, which controls respiration and circulation; and the periaqueductal grey, which is important for various autonomic functions such as circulation, respiration, and thermoregulation [4,8]. Autonomic dysfunction and excessive sympathetic activity are more common following brainstem stroke. The heart rate (HR) is regulated by the autonomic nervous system, whereby the sympathetic part leads to an increase in HR and a decrease in heart rate variability (HRV), while the parasympathetic part does exactly the opposite [3], thereby changes in HRV reflect the overall level of ANS dysfunction [9,10].

HRV, the beat-to-beat variance of HR, can be assessed using time domain or frequency domain analysis [9,11]. Time domain indices of HRV quantify the amount of variability in measurements of the interval between beats, thereby the standard deviation (SD) of the heartbeat intervals describes the dispersion around the mean and is influenced by both the sympathetic and parasympathetic nervous system. The coefficient of variation (CV) as a relative measure describes the dispersion around the mean. The Successive Variability (SV) reflects the fluctuations from one beat to the next and is primarily used to estimate the vagally mediated changes of the HR [10,11,12]. Frequency domain measurements estimate the distribution of absolute or relative power into four frequency bands. These are essentially the ultra-low-frequency (ULF) band, the very-low frequency (VLF) band, the low-frequency (LF) band associated with sympathetic as well as parasympathetic activities, the high-frequency (HF) band associated mainly with parasympathetic activity, and the LF/HF ratio revealing sympathetic or parasympathetic dominance [10,11,12]. Primary brain injury from ICH may cause secondary injuries via cellular changes or brain edema affecting autonomic regulation, often leading to sympathetic hyperactivity [13]. Since these damages are likely to affect the above-mentioned brain regions involved in autonomic regulation, ICH is often complicated by dysfunction of the ANS and sympathetic–parasympathetic imbalance, primarily manifested as elevated activity of the sympathetic nervous system (sympathetic hyperactivity) [4,9,12,14,15,16].

Autonomic dysfunction has been linked to adverse outcomes following ICH [9,14,15,17]. HRV showed significant impairment within the first 14 days post-ICH compared to matched controls, correlating independently with poor neurological outcomes at 3-month follow-up [9]. A post hoc analysis of an ATACH-2 (Antihypertensive Treatment in Intracerebral Hemorrhage 2) trial further confirmed that average real variability (ARV) of HRV within the first 24 h independently predicted hematoma expansion and unfavorable outcomes at follow-up [15]. In another study, a higher SD of HRV in the acute first 24 h post-ICH was associated with a worse outcome at 3 months, with factors such as intraventricular hemorrhage (IVH), prolonged QRS complex > 120 ms, and female gender also associated with higher HRV [18]. Also, in another study, higher CV of HRV and higher mean HR during the first 72 h post-ICH were associated with increased risk of unfavorable functional outcomes and mortality [19]. A comprehensive systematic review involving 19 studies affirmed the association of a higher HRV with poor functional outcome and increased mortality in both ICH and subarachnoid hemorrhage (SAH) [12]. Moreover, HRV, particularly low- and high-frequency power bands, was significantly lower in ICH patients compared to controls, thereby these autonomic parameters predicted poor outcome independently and correlated with stroke severity and hemorrhage volume [17,20]. Heart rate entropy quantifying the irregularity and complexity of heart beat intervals over time was identified as a strong predictor of mortality in ICH patients and could, combined with the ICH score, increase the predictive performance for mortality [14]. Furthermore, HRV has emerged as a potential marker for autonomic dysfunction and a predictor of subsequent fever development in ICH patients [21].

Summing up, variations and complexities in HR and HRV, measured through various statistical methods, represent significant indicators of outcome in ICH patients and therefore are a promising avenue to identify those patients who might benefit from targeted neuromodulation and nuanced management strategies.

Our study seeks to investigate HRV as a parameter of autonomic dysfunction and its correlation with functional outcomes in a well-described ICH cohort leveraging Big Data analytics and continuous heart rate values.

## 2. Materials and Methods

### 2.1. Study Design

In this observational cohort study, prospectively collected data are analyzed in a retrospective manner.

### 2.2. Study Population and Exclusion Criteria

The study population comprised patients from our in-house stroke registry, specifically focusing on patients with the diagnoses of spontaneous ICH (diagnosis code I61 of the International Classification of Diseases, 10th edition) who were admitted to the neurological intensive care unit (NICU) and/or stroke unit (SU) of the University Hospital of Tübingen between 1 December 2015 and 31 December 2020.

The inclusion criteria were as follows: (1) age > 18 years and (2) cerebral imaging showing ICH. Exclusion criteria included the following: (1) duration of hospital stay < 24 h; (2) neurosurgical intervention immediately after baseline-CT; (3) traumatic ICH; (4) intracranial tumor as the etiology for ICH; (5) hemorrhagic transformation of acute ischemic stroke (AIS); (6) patients with withdrawal of medical management at admission or within the first 24 h; and (7) incomplete data on functional outcome at 3-month follow-up. The cohort has been previously described in detail elsewhere [22].

The clinical severity of ICH was assessed using the National Institutes of Health Stroke Scale (NIHSS) [23]. The ICH of the patients were classified using the SMASH-U and CLAS-ICH systems [24,25]. The ICH score, which includes the Glasgow Coma Scale (GCS), age, intraventricular hemorrhage (IVH), and hematoma volume, was used to assess the prognostic 30-day mortality. The ICH score ranges from 0 to 6, with higher scores indicating greater severity and worse prognosis [26]. Patients’ clinical characteristics were extracted from the EHR. Patients aged 55 years or younger were considered to have juvenile stroke [27].

### 2.3. Cerebral Imaging

Each patient received initial CT imaging at admission, which included non-contrast CT and CT angiography (CTA) according to in-house standard operation procedures (SOP). Additionally, all participants underwent follow-up brain imaging (either CT or magnetic resonance imaging (MRI)) 18–24 h after admission. An independent, clinically experienced neuroradiologist assessed all images in a blinded and randomized fashion. White matter disease was evaluated using the age-related white matter changes rating scale (Fazekas (Fz) score) [28]. The volume of ICH was quantitatively assessed using the ABC/2 formula [29,30]. Early hematoma expansion was defined either as absolute [≥6 mL] or relative [≥33%] change of ICH volume [29,30].

### 2.4. Continuous Monitoring of Heart Rate (HR) and Calculation of Variabilities

Upon admission to the neurological intensive care unit (neuro ICU), high-fidelity and real-time vital monitoring of the patients was initiated, including HR monitoring. Detailed monitoring protocols are provided elsewhere [22]. Data from this continuous recording were systematically retrieved from our electronic health records (EHR) with the clinical information system Intellispace Critical Care and Anesthesia information system (ICCA), Philips Healthcare. All available measurements of HR for the first 96 h after admission were evaluated for different time intervals after admission, mainly 0–2 h, 0–8 h, 0–12 h, 0–24 h, 0–48 h, 0–72 h, and 8–16 h, 16–24 h, 24–48 h, 48–72 h, 72–96 h post-admission. The following HRV indices were calculated: (1) mean, (2) standard deviation (SD), (3) coefficient of variation (CV, CV = SD/mean × 100), and (4) successive variation (SV), representing the averaged squared difference between the consecutive order of individual measurements [10,11,12]:

$$\sqrt{\frac{1}{n-1}\sum_{i=1}^{n-1}{\left(X_{i+1}-X_{i}\right)}^{2}}$$

Patients were divided into low vs. high HRV groups based on whether their HRV SD over the first 72 h was below or above the average HRV SD of 11.4 bpm, derived from the overall cohort distribution. Quartiles of HRV SD were calculated, and the interquartile range (IQR) was used to determine the threshold for low and high HRV groups. Specifically, the threshold was set at the median HRV SD, with patients falling below the first quartile classified as low HRV and those above the third quartile classified as high HRV [31].

### 2.5. Clinical Outcomes

Functional outcome was assessed using the modified Rankin Scale (mRS). The follow-up was assessed at discharge and at follow-up after 90 days, either by telephone calls or outpatient visits. Clinical outcome was assumed excellent if mRS was 0–1 and good if mRS was 0–2 [32]. Further outcome variables included mortality, in-hospital complications such as infections or delirium, and need for interventions like extraventricular ventricular drainage (EVD), medications like catecholamine or beta-blocker administration.

### 2.6. Study Endpoints

The primary endpoint of this study was to assess the impact of HRV, expressed as SV, SD, and CV within different time intervals post-admission, on good outcome at follow-up after 90 days. Secondary endpoints included the effect of HRV on HE, outcome at discharge, mortality at discharge and follow-up, and the influence of patient characteristics (e.g., presence of AF, beta-blocker treatment) as well as ICH characteristics (e.g., localization, size) on HRV changes.

### 2.7. Statistical Analysis

The successively recorded HR values were determined using the programming language R (current version, R 4.2.0). Data quality was ensured by cleaning and preprocessing, including handling missing values and outliers for the continuous HR values. The data were transformed into a format suitable for analysis in the spreadsheet software Excel version 16.67 (Microsoft, Redmond, WA, USA). Statistical analysis was carried out using SPSS version 29.0.2. (IBM, Armonk, NY, USA). Baseline characteristics, outcome parameters as well as patient and bleeding characteristics were analyzed. Continuous variables were presented as mean ± standard deviation (SD), ordinal variables as median with interquartile range (IQR), and categorical variables as total number (n) and percentage (%). This type of data description is suitable if the values are normally distributed, which was confirmed using the Shapiro–Wilk test. Differences between group characteristics were examined using *t*-tests for continuous variables, Pearson’s chi-squared test for categorical variables, and the Wilcoxon rank-sum test for ordinal variables. Logistic regression analysis was performed, including SD of HRV and mean HR in the 0–72 h interval and other predictors plausibly associated with outcome, to evaluate their correlation with functional outcome at 90 days follow-up. Statistical significance was set at *p* < 0.05.

### 2.8. Ethics Statement

Our study was approved by the local ethics committee (protocol 545/2022BO2) and was performed in accordance with the ethical standards of the 1964 Declaration of Helsinki and all later amendments. Participants’ individual informed consent was waived due to the clinic-wide consent policy, which allows the use of de-identified routine treatment data for research purposes.

## 3. Results

### 3.1. Baseline Characteristics and Functional Outcome at Follow-Up after 90 Days

A total of 261 ICH patients (mean age ± SD 69.9 ± 16.5 years; 47.9% female) with moderate to severe ICH (median NIHSS 6 (2, 12), median ICH-score 1 (0, 2)) were analyzed. Good outcome at follow-up was observed in 106 patients (40.6%). Of the cohort, 54% (n = 141) had low HRV (SD ≤ 11.4 ms), while 118 had high HRV (SD > 11.4 ms).

No significant differences were found between the low and high HRV groups regarding age (mean age 70.9 ± 14.7 years vs. 68.1 ± 15.6 years, *p* = 0.143) or sex (51.1% female vs. 44.9% female, *p* = 0.326). Other baseline characteristics, including premorbid mRS (median 0 (0, 2) in both groups, *p* = 0.961), NIHSS at admission (median 5 (2, 10) in the low HRV group vs. 7 (4, 12) in the high HRV group, *p* = 0.063), GCS at admission (median 15 (12, 15) in the low HRV group vs. 14 (11, 15) in the high HRV group, *p* = 0.253), and ICH score (median 1 (0, 2) in both groups, *p* = 0.626) also showed no significant differences.

Patients with low HRV had a significantly lower NIHSS at 24 h (median 5 (2, 11)) compared to those with high HRV (median 8 (4, 14)) (*p* = 0.018). Other notable differences included lower mean arterial pressure (MAP) (107.5 ± 22.0 vs. 115.9 ± 23.7 mmHg, *p* = 0.005) and lower mean HR at admission (74.7 ± 13.3 vs. 85.1 ± 18.4 bpm, *p* < 0.001) in the low HRV group compared to the higher HR high HRV group. Deep ICH localization was less common in the low HRV group compared to the high HRV group (41.1 vs. 57.6%, *p* = 0.008). Regarding complications, chronic renal failure was less common in the low HRV group (7.1 vs. 16.9%, *p* = 0.018). At discharge, the low HRV group had a lower discharge NIHSS (median 4 (1, 9)) than the high HRV group (median 7 (2, 11)) (*p* = 0.048). The median mRS at discharge was also lower in the low HRV group (4 (2, 5)) compared to the high HRV group (4 (3, 5)) (*p* = 0.003). Additionally, a higher percentage of patients with low HRV achieved a good outcome at discharge (27.7%) compared to those with high HRV (14.4%) (*p* = 0.010). At 90-day follow-up, more patients with low HRV had a good outcome compared to those with high HRV (46.1% vs. 33.9%, *p* = 0.047). However, the mortality rate at 90 days was numerically but not significantly different (14.2% vs. 10.2%, *p* = 0.330). For further details, see Table 1.

### 3.2. Longitudinal Analysis

**Figure 1:**
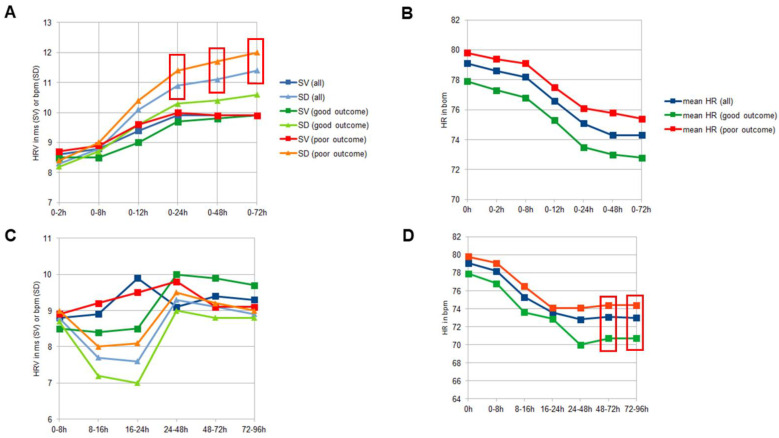
HRV (expressed in SV and SD) and HR over different time periods. (**A**): Longitudinal analysis of HRV in continuous intervals. All patients (blue) and those with a good (green) or poor (red) functional outcome after 90 days are shown. (**B**): Longitudinal analysis of mean HR in continuous intervals. (**C**): Longitudinal analysis of HRV in successive intervals. (**D**): Longitudinal analysis of mean HR in successive intervals. Red boxes show the significant different values. Abbreviations are HRV = heart rate variability, HR = heart rate, SD = standard deviation, SV = successive variability.

In all patients, HRV was lowest at admission (0–2 h: HRV SD 8.3 ± 4.5 bpm) and increased thereafter. When examining continuous time intervals, HRV showed a continuous increase (0–72 h: HRV SD 11.4 ± 3.9 bpm). However, when divided into successive time periods, HRV increased only in the first 24 to 48 h with subsequent leveling off to a value range slightly below the maximum HRV (48–72 h: 9.1 ± 4.0 bpm). Conversely, mean HR was highest at admission (0 h: HR 79.1 ± 16.5 bpm) and decreased thereafter, with the most significant decrease in the first 24 h (0–24 h: 75.1 ± 12.0 bpm) (see Figure 1).

### 3.3. HRV and HR and Further Course

Patients with good outcomes had less variability in HRV in the first three days compared to those with poor outcomes. Specifically, SD and CV values were lower in the good outcome group. (e.g., 0–72 h: HRV SD good outcome 10.6 ± 3.5 bpm vs. poor outcome 12.0 ± 4.0 bpm; *p* = 0.004). Mean HR also differed significantly after two days, with lower HR in patients with good outcomes (e.g., 48–72 h: mean HR good outcome 70.7 ± 11.0 bpm vs. poor outcome 74.4 ± 13.0 bpm; *p* = 0.034). There was no significant difference in HRV or HR between patients with and without hematoma expansion within the first 24 h (see Table 2).

### 3.4. HRV and AF

Patients without AF showed similar HRV patterns to the overall cohort, with lower HRV in the good outcome group (e.g., 0–24 h: HRV SD good outcome 9.7 ± 3.5 bpm vs. poor outcome 11.5 ± 4.2 bpm; *p* = 0.002). On the contrary, those patients with AF and good outcome had higher HRV from the mean (e.g., 0–24 h: HRV SD good outcome 13.6 ± 4.8 bpm vs. poor outcome 11.0 ± 4.1 bpm; *p* = 0.048) and on top of that greater HR fluctuations in the first three days (e.g., 0–24 h: HRV SV good outcome 14.6 ± 7.7 ms vs. poor outcome 9.8 ± 3.7 ms; *p* = 0.001) (see Supplemental Table S1). Patients with AF had higher mean HR throughout the first 96 h compared to those without AF (e.g., 0–12 h: mean HR with AF 78.2 ± 14.5 bpm vs. no AF 76.1 ± 11.4; *p* = 0.012). Additionally, variability from the mean HR was higher in patients with AF (e.g., 0–12 h: HRV SD with AF 10.6 ± 4.7 bpm vs. 9.9 ± 4.5 bpm; *p* = 0.038). No significant difference in HRV indices or mean HR was observed between patients treated with and without beta-blockers.

### 3.5. HRV and Patient Characteristics (Sex and Age)

Women had lower HRV from the mean in specific time intervals. Specifically, in the 0–24 h interval, male patients had significantly higher SD (*p* = 0.037). This trend continued in the 0–48 h interval, where SD (*p* = 0.008) and CV (*p* = 0.040) were significantly higher in males. By the 0–72 h interval, SD (0–72 h: HRV SD women 11.3 ± 3.8 bpm vs. men 11.5 ± 3.9 bpm; *p* = 0.004) and CV (*p* = 0.018) remained significantly higher in males. In other specific intervals like 8–16 h and 16–24 h, no significant differences in HR parameters were observed between genders. However, in the 48–72 h interval, the mean HR was significantly lower in males (*p* = 0.034), and in the 72–96 h interval, the mean HR was slightly higher in males (*p* = 0.041). Furthermore, women had higher mean HR from the third day onwards (48–72 h: mean HR women 73.5 ± 11.5 bpm vs. men 72.7 ± 13.2 bpm; *p* = 0.034) compared to men. At the 0–24 h interval, HR SD showed a significant difference (*p* = 0.037), with males having a slightly higher HR SD, while other parameters showed no significant differences (*p* = 0.090 to 0.675). In the 0–48 h interval, HR SD (*p* = 0.008) and HR CV (*p* = 0.040) were significantly higher in males, while other parameters were not significantly different (*p* = 0.063 to 0.834) (see Supplemental Table S2). With regard to age, patients with juvenile stroke, up to the age of 55 years, had generally higher HRV, whereby the difference was significant from the third day onwards (48–72 h: HRV SD ≤55 years 10.3 ± 5.2 vs. >55 years 8.8 ± 3.7 bpm; *p* = 0.018 and HRV SV ≤55 years 11.7 ± 8.3 ms vs. >55 years 9.0 ± 4.1 ms; *p* = 0.002). Also, the mean HR tended to be higher in younger patients.

### 3.6. HRV and Bleeding Characteristics

Considering the ICH localization, in contrast to infratentorial ICH, patients with supratentorial ICH had a significantly different mean HR over the whole examined first 96 h after admission to the hospital, thereby patients with deep ICH had the lowest HF values and the difference was more pronounced in the later time intervals (e.g., 24–48 h: mean HR deep 69.4 ± 10.9 bpm vs. average all localizations 72.8 ± 13.3 bpm; *p* = 0.003), whereas those with lobar ICH had rather higher HR values and the difference was more pronounced in the beginning (e.g., 0–12 h: mean HR lobar 78.2 ± 12.7 ms vs. average all localizations 76.6 ± 12.3 ms; *p* = 0.039). During the first three days, patients with brainstem ICH showed significantly lower HR fluctuations from one time point to the next (0–72 h: HRV SV brainstem 7.5 ± 2.2 ms vs. average all localizations 9.9 ± 4.3 ms; *p* = 0.022). In contrast, patients with lobar ICH had higher HR fluctuations from one time point to the next during the same intervals (0–72 h: HRV SV lobar 10.5 ± 4.82 ms vs. average all localizations 9.9 ± 4.3 ms; *p* = 0.038). Considering the bleeding size, ICH volume regarding small (<30 mL) and large (≥30 mL) ICH had no significant influence on all HRV indices and in the mean HR.

### 3.7. Univariate Analysis of HR and HRV Based on Functional Outcome after 90 Days

Univariate analysis confirmed that reduced variability in HRF from the mean was significantly associated with good functional outcomes at 90-day follow-up, as expressed by SD (0–24 h OR 0.934; CI 0.876; *p* = 0.039; 0–48 h OR 0.910; CI 0.848–0.976; *p* = 0.009; 0–72 h OR 0.903; CI 0.841–0.969; *p* = 0.005) and CV (0–48 h OR 0.005; CI 0.000–0.834; *p* = 0.042; 0–72 h OR 0.002; CI 0.000–0.383; *p* = 0.020). Reduced mean HR at day 3 and 4 (48–72 h OR 0.975; CI 0.953–0.998; *p* = 0.035; 72–96 h OR 0.974; CI 0.949–0.999; *p* = 0.043) also showed a significant correlation with good functional outcome. For further details, see Table 3.

### 3.8. Multivariate Analysis of 0–72 h HRV and Mean HR Based on Functional Outcome after 90 Days

**Figure 2:**
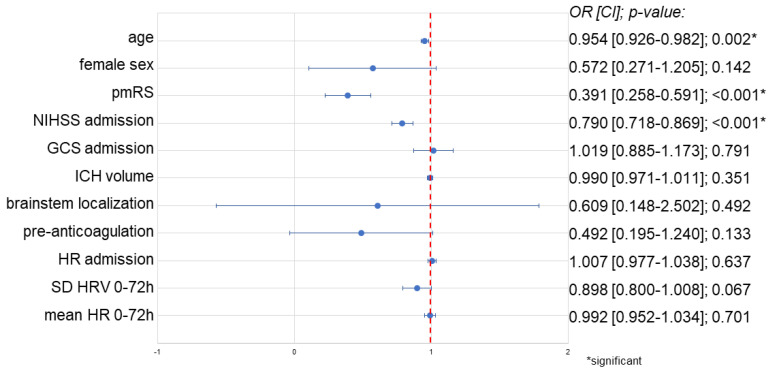
Logistic regression based on good functional outcome (mRS) at 90 days follow-up. Multivariate analysis of 0–72 h HRV and mean HR based on functional outcome after 90 days. ICH volume is given in cm3. Abbreviations: CI = confidence interval, GCS = Glasgow Coma Scale, HR = heart rate, HRV = heart rate variability, ICH = intracerebral hemorrhage, NIHSS = National Institutes of Health Stroke Scale, OR = odds ratio, SD = standard deviation.

Multivariate logistic regression analysis identified age (OR 0.954; CI 0.926–0.982; *p* = 0.002), high pmRS (OR 0.391; CI 0.258–0.591; *p* < 0.001), and high NIHSS at admission (OR 0.790; CI 0.718–0.869; *p* < 0.001) as significant predictors of poor functional outcome at follow-up. Reduced SD of HR in the 0–72 h time interval showed a non-significant trend towards a good outcome at follow-up (OR 0.898; CI 0.800–1.008; *p* = 0.067). Other factors, including female sex, GCS at admission, anticoagulation at admission, HR at admission, ICH volume (in cm^3^), brainstem bleeding localization, and mean HR in the time interval 0–72 h were not associated with functional outcome (see Figure 2).

## 4. Discussion

In this study, which included 261 patients with spontaneous ICH, we aimed to analyze the influence of HRV on functional outcome after 90 days using continuous heart rate monitoring and Big Data analytics. The main findings are as follows:

Firstly, all ICH patients initially experienced a restriction in HRV, which then increased steadily over the first two to three days before leveling off. Our results support the findings of previous studies, that described autonomic dysregulation with sympathetic overactivation in the context of ICH [3,4,9,14,17]. Sympathetic nervous system dominance is known to be associated with reduced HRV and increased absolute HR [12,15]. In contrast to the study by Qu et al., our patients experienced an increase in HRV within the first few days, while HRV remained impaired in the patients examined in the aforementioned study [9].

Secondly, patients with good functional outcome had more strongly reduced HRV than those with poor outcome. Previous studies showed similar results: the post-hoc analysis of the ATACH-2 trial revealed that both an elevated mean HR and increased HR average real variability within the first 24 h after admission were independently linked to poor functional outcomes at 90 days follow-up in patients with acute ICH, as well as with hematoma expansion after 24 h [16].

However, we did not find any significant differences in HR or HRV in relation to HE within 24 h in our cohort. Both our study and the post-hoc analysis of ATACH-2 focused on time–domain parameters of HRV, such as SD, CV, and SV. Marino et al. conducted a systematic review of 19 studies, which found that higher HRV was consistently associated with poor functional outcome in both ICH and SAH [12]. Overall, these results suggest an initial protective effect of sympathetic hyperactivity on functional outcomes after ICH. We also investigated patients with atrial fibrillation (AF) concerning the effects of HRV on functional outcomes. So far, it has been described that a higher HRV increases the risk of developing AF [33]. In addition, patients with permanent AF appear to have a higher HRV than those with paroxysmal AF [33]. In addition, patients with permanent AF appear to have a higher HRV than those with paroxysmal AF [33]. Interestingly, AF patients with good outcomes had higher HRV and greater HR fluctuations in the first few days. This might be due to pre-existing heart disease associated with AF, which can impair HRV and increase the risk of coronary ischemia, cardiac arrhythmias, myocardial injury, and sudden cardiac death [4,34]. Apart from lower HRV in the first three days, patients with good outcomes also had lower average HRF from the third day onwards. Since a lower HR is associated with lower sympathetic activity [3], this result implies a worsening of the outcome prognosis in the case of persistent sympathetic hyperactivity. Overall, our results indicate a short-term positive effect of sympathetic hyperactivity in the initial stage, manifested by a comparatively reduced HRV, whereas a persistence of sympathetic hyperactivity, manifested by an elevated absolute mean HR, longer than three days, seems to have a negative effect and is associated with a poorer functional outcome. This fits with the already established knowledge that catecholamines, which are elevated during sympathetic activation, are important in the short term for survival in emergency situations, but have a harmful effect over a longer period of time [35]. This is further demonstrated by the impact of hyperglycemia, which worsens brain edema and increased perihematomal cell death, resulting in poorer functional outcomes for ICH patients.

Additionally, it is associated with higher mortality rates and poor functional outcomes, significantly impacting prognosis. Hyperglycemia in ICH is often induced by the activation of the sympathetic nervous system, which increases catecholamine release, enhances insulin resistance, and triggers stress-induced cytokine production, thereby elevating blood glucose levels. Effective management of sympathetic activity is essential to control hyperglycemia in such acute conditions [36].

Thirdly, regarding the influence of patients and hemorrhage characteristics on HRV, our results showed that HRV was higher in younger patients, which has already been described [3]. This could be due to the fact that the functional capacity of the parasympathetic branch of the autonomic nervous system in particular tends to decline with age [37]. Patients with brainstem or deep ICH had initially lower HRV, likely due to the critical role of the brainstem and hypothalamus in autonomic regulation [4]. An impairment of HRV when these structures are affected therefore seems quite plausible. Patients with AF had higher absolute HR, with HRV varying over time. However, this finding per se would already be well explained by the AF itself and does not necessarily have to be due to a dysfunction of the CAN. No significant difference in HRV or HR was observed between patients treated with beta-blockers and those who were not. However, there are different reports regarding treatment with beta-blockers for the treatment of sympathetic hyperactivity. In a study by Zhang et al., ICH mice treated with metoprolol had improved cardiac and neurological function, and they attributed this to the fact that the suppression of sympathetic overactivity by metoprolol attenuated cardiac inflammation, fibrosis, and oxidative stress after ICH [38]. In contrast, in the analysis by Shoup et al., beta-blockers were not associated with any class-specific survival benefit in acute ICH [39], which is consistent with our result. Significant differences in HRV between males and females were observed in the 24–48 h, 48–72 h, and 72–96 h intervals, indicating potential gender-specific variations in HR response post-ICH. Sex-related differences in HRV among ICH patients are evident in several aspects. Female ICH patients tend to exhibit greater vagal activity, reflected in higher high-frequency (HF) power of HRV, and lower low-frequency (LF) power, resulting in a lower LF/HF ratio compared to male patients [40]. These sex-related differences in autonomic regulation may contribute to different outcomes post-ICH. Studies indicate that female patients often have higher HRV in the acute phase after ICH, which is associated with poorer outcomes [18]. Furthermore, women generally present with worse functional outcomes and higher mortality rates post-ICH compared to men, potentially due to these differences in autonomic regulation and higher HRV [41]. Additionally, sex differences in HRV can be influenced by hormonal factors, with studies showing that estrogen may have a protective effect on autonomic regulation, enhancing vagal activity in women [42].

One notable strength of our study is the well-defined cohort of ICH patients. On the one hand, this proved statistical power and therefore differences can be better detected. On the other hand, it also provides high validity and thus the results can be better transferred to the general population. Additionally, the use of Big Data leveraging all available data from the individual patients using EHR and detailed continuous HR monitoring from admission improved the reliability of our HRV analysis. The use of a comprehensive set of indices (SV, SD, CV), ensured the robustness of our results. On the other hand, our study has several limitations. First of all, it is a retrospective analysis of prospectively collected data. This included potential biases inherent in its retrospective design. One of the biggest strengths of our study leveraging all available data from the individual patients also presented a major limitation, as the calculation of HRV was based on HR values stored during monitoring, rather than individual interbeat intervals. Consequently, our data could not be used to calculate classic indices of HRV, such as SD of NN intervals (SDNN), root mean square of successive RR interval differences (RMSSD), or percentage of successive RR intervals that differ by more than 50 ms (pNN50). According to the respective calculation methods, however, the SD we used is comparable with SDNN, and the SV is comparable with RMSSD. Furthermore, the influence of other cofactors associated with the outcome, despite being considered in the statistical testing, probably cannot be completely eliminated. Additionally, the exclusion of patients who required immediate neurosurgical intervention after CT imaging may have influenced the statistical significance of the HRV results.

## 5. Conclusions

In conclusion, our study confirms that spontaneous ICH is associated with autonomic dysregulation and sympathetic hyperactivity, leading to impaired HRV. Initial sympathetic dominance appears protective, while prolonged sympathetic hyperactivity beyond three days seems detrimental to functional outcomes. Further prospective studies are needed to pinpoint the transition from protective to harmful sympathetic activity. Since sympathetic activity is generally discordant to HRV and concordant to HR, HRV and HR can be used to monitor autonomic dysregulation in ICH patients. However, there are currently no data on the optimal value of HRV. Therefore, the identification of optimal HRV values for clinical practice need further investigation. Identifying prolonged HRV suppression or increased HR could help initiate countermeasures, such as targeted neuromodulation, to mitigate sympathetic hyperactivity. The role of beta-blockers in this context remains controversial in view of our results and the literature and warrants further research.

## Figures and Tables

**Table 1:** Characteristics and outcome of patients with ICH, based on low or high SD of heart rate variability in 0–72 h (≤ or > mean of 11.4 bpm).

	All ICH-Patients (n = 261)	ICH-Patients with SD of HRV 0–72 h ≤ Mean of 11.4/min (n = 141)	ICH-Patients with SD of HRV 0–72 h > Mean of 11.4/min (n = 118)	*p*-Value
**general**				
age (y), mean ± SD	69.6 ± 15.2	70.9 ± 14.7	68.1 ± 15.6	0.143
sex-female, n (%)	127 (48.7%)	72 (51.1%)	53 (44.9%)	0.326
pmRS, median (IQR)	0 (0, 2)	0 (0, 2)	0 (0, 1)	0.961
**admission**				
NIHSS, median (IQR)	6 (2, 12)	5 (2, 10)	7 (4, 12)	0.063
GCS, median (IQR)	14 (11, 15)	15 (12, 15)	14 (11, 15)	0.253
ICH-score, median (IQR)	1 (0, 2)	1 (0, 2)	1 (1, 2)	0.626
**after 24 h**				
NIHSS, median (IQR)	6 (2, 13)	5 (2, 11)	8 (4, 14)	0.018
**discharge**				
NIHSS, median (IQR)	5 (1, 11)	4 (1, 9)	7 (2, 11)	0.048
mRS, median (IQR)	4 (3, 5)	4 (2, 5)	4 (3, 5)	0.003
good outcome, n (%)	57 (21.8%)	39 (27.7%)	17 (14.4%)	0.010
mortality, n (%)	9 (3.4%)	4 (2.8%)	5 (4.2%)	0.542
**follow-up after 90 days**				
mRS, median (IQR)	3 (1, 5)	3 (1, 4)	3 (1, 5)	0.110
good outcome, n (%)	106 (40.6%)	65 (46.1%)	40 (33.9%)	0.047
mortality, n (%)	32 (12.3%)	20 (14.2%)	12 (10.2%)	0.330
**risk factors**				
arterial hypertension, n (%)	211 (80.8%)	112 (79.4%)	97 (82.2%)	0.574
diabetes mellitus, n (%)	49 (18.8%)	22 (15.6%)	27 (22.9%)	0.143
atrial fibrillation, n (%)	67 (25.7%)	34 (24.1%)	33 (28.0%)	0.485
hypercholesterinemia, n (%)	51 (19.5%)	28 (19.9%)	23 (19.5%)	0.941
smoking, n (%)	37 (14.2%)	20 (14.2%)	17 (14.4%)	0.960
alcohol, n (%)	21 (8.0%)	9 (6.4%)	12 (10.2%)	0.277
obesity (BMI > 30), n (%)	56 (21.6%)	28 (19.9%)	28 (23.7%)	0.456
coronary heart disease, n (%)	42 (16.1%)	23 (16.3%)	19 (16.1%)	0.964
chronic renal failure, n (%)	30 (11.5%)	10 (7.1%)	20 (16.9%)	0.017
chronic hepatic failure, n (%)	5 (1.9%)	2 (1.4%)	3 (2.5%)	0.525
malignancy, n (%)	13 (5.0%)	11 (7.8%)	2 (1.7%)	0.018
CHA2DS2-VaSc-Score, median (IQR)	3 (2, 4)	4 (2, 4)	3 (2, 4)	0.729
**premedication at admission**				
anticoagulation, n (%)	65 (24.9%)	36 (25.5%)	29 (24.6%)	0.860
antiplatelet therapy, n (%)	60 (2.0%)	37 (26.2%)	23 (19.5%)	0.197
**laboratory findings at admission**				
INR, mean ± SD	1.1 ± 0.4	1.1 ± 0.3	1.2 ± 0.4	0.289
PTT (s), mean ± SD	24.6 ± 5.3	24.5 ± 5.1	24.9 ± 5.5	0.606
thrombocyte count, mean ± SD	216 ± 79	216.9 ± 76.6	215.8 ± 83.7	0.912
**vital signs at admission**				
MAP (mmHg), mean ± SD	111.2 ± 23.2	107.5 ± 22.0	115.9 ± 23.7	0.005
HR (bpm), mean ± SD	79.1 ± 16.5	74.7 ± 13.3	85.1 ± 18.4	<0.001
**imaging**				
deep localization, n (%)	127 (48.7%)	58 (41.1%)	68 (57.6%)	0.008
lobar localization, n (%)	101 (38.7%)	60 (42.6%)	40 (33.9%)	0.155
brainstem localization, n (%)	16 (6.1%)	10 (7.1%)	6 (5.1%)	0.506
cerebellar localization, n (%)	28 (10.7%)	16 (11.3%)	12 (10.2%)	0.762
IVH primary, n (%)	1 (0.4%)	1 (0.7%)	0 (0.0%)	0.319
IVH secondary, n (%)	92 (35.2%)	48 (34.0%)	44 (37.3%)	0.554
superficial siderosis, n (%)	21 (8.0%)	11 (7.8%)	10 (8.5%)	0.845
FAZEKAS PWM, median (IQR)	1 (0, 1)	1 (0, 2)	1 (0, 1)	0.329
FAZEKAS DWM, median (IQR)	1 (0, 2)	1 (0, 2)	0 (0, 2)	0.568
ICH Volume (mL), mean ± SD	17.4 ± 24.6	16.3 ± 24.2	18.3 ± 24.6	0.655
early hematoma expansion, n (%)	37 (14.2%)	17 (12.1%)	19 (16.1%)	0.356
**etiology SMASH-U**				0.076
structural vascular lesion, n (%)	27 (10.3%)	18 (12.8%)	9 (7.6%)	
systemic/other disease, n (%)	13 (5.0%)	7 (5.0%)	6 (5.1%)	
medication, n (%)	52 (19.9%)	31 (22.0%)	21 (17.8%)	
amyloid angiopathy, n (%)	23 (8.8%)	14 (9.9%)	9 (7.6%)	
hypertension, n (%)	111 (42.5%)	54 (38.3%)	56 (47.5%)	
undetermined, n (%)	35 (13.4%)	17 (12.0%)	17 (14.4%)	
**etiology CLAS-ICH subtypes, n (%)**				0.055
well defined (grade 1), n (%)	110 (42.1%)	68 (48.2%)	42 (35.6%)	
A1, n (%)	56 (21.5%)	34 (24.1%)	22 (18.6%)	
C1, n (%)	18 (6.9%)	12 (8.5%)	6 (5.1%)	
S1, n (%)	35 (13.4%)	22 (15.6%)	13 (11.0%)	
possible (grade 2), n (%)	74 (28.4%)	40 (28.4%)	34 (28.8%)	
A2, n (%)	93 (35.6%)	47 (33.3%)	46 (39.0%)	
C2, n (%)	11 (4.2%)	4 (2.8%)	7 (5.9%)	
M2, n (%)	19 (7.3%)	8 (5.7%)	11 (9.3%)	
S2, n (%)	6 (2.3%)	3 (2.1%)	3 (2.5%)	
multiple (≥2 grades 1 or 2), n (%)	7 (2.7%)	2 (1.4%)	5 (4.2%)	
insufficient work-up (grade 9), n (%)	70 (26.8%)	31 (22.0%)	37 (31.4%)	
**Intervention**				
any procoagulation/proclotting medication, n (%)	73 (28.0%)	38 (27.0%)	34 (28.8%)	0.741
platelet concentrate, n (%)	15 (5.7%)	6 (4.3%)	9 (7.6%)	0.260
minirin, n (%)	15 (5.7%)	9 (6.4%)	5 (4.2%)	0.441
PCC, n (%)	40 (15.3%)	24 (17.0%)	16 (13.6%)	0.441
andexanet alpha, n (%)	12 (4.6%)	4 (2.8%)	8 (6.8%)	0.148
idarucizumab, n (%)	1 (0.4%)	1 (0.7%)	0 (0.0%)	0.319
vitamin K, n (%)	7 (2.7%)	3 (2.1%)	4 (3.4%)	0.543
protamine, n (%)	3 (1.1%)	2 (1.4%)	1 (0.8%)	0.663
neurosurgical intervention, n (%)	32 (12.3%)	15 (10.6%)	16 (13.6%)	0.477
EVD, n (%)	36 (13.8%)	20 (14.2%)	16 (13.6%)	0.885
intubation, n (%)	96 (36.8%)	58 (41.1%)	37 (31.4%)	0.144
**complications**				
infection, n (%)	137 (52.5%)	70 (49.6%)	66 (55.9%)	0.315
delirium, n (%)	103 (39.5%)	49 (34.8%)	54 (45.8%)	0.073
**length of stay**				
ICU (days), mean ± SD	9.9 ± 9.3	8.9 ± 9.7	11.0 ± 8.7	0.068
hospital (days), mean ± SD	14.6 ± 10.4	14.5 ± 11.7	14.8 ± 8.6	0.860
**medication**				
antibiotics, n (%)	158 (60.5%)	82 (58.2%)	75 (63.6%)	0.376
benzodiazepines, n (%)	75 (28.7%)	28 (19.9%)	47 (39.8%)	<0.001
anticonvulsants, n (%)	62 (23.8%)	34 (24.1%)	28 (23.7%)	0.943
antipsychotics, n (%)	102 (39.1%)	44 (31.2%)	58 (49.2%)	0.003
antidepressants, n (%)	50 (19.2%)	20 (14.2%)	30 (25.4%)	0.025
melatonin, n (%)	102 (39.1%)	59 (41.8%)	43 (36.4%)	0.376
NSAIDs, n (%)	58 (22.2%)	31 (22.0%)	26 (22.0%)	0.993
pyrazolones, n (%)	207 (79.3%)	113 (80.1%)	93 (78.8%)	0.793
opioids I, n (%)	24 (9.2%)	11 (7.8%)	13 (11.0%)	0.383
opioids II, n (%)	144 (55.2%)	69 (48.9%)	75 (63.6%)	0.018
a2-agonists, n (%)	131 (50.2%)	56 (39.7%)	75 (63.3%)	<0.001
ace-inhibitors, n (%)	147 (56.3%)	73 (51.8%)	74 (62,7%)	0.063
beta-blockers, n (%)	133 (51.0%)	70 (49.6%)	63 (53.4%)	0.550
potassium channel-blockers, n (%)	13 (5.0%)	8 (5.7%)	5 (4.2%)	0.595
angiotensin1-antagonists, n (%)	71 (27.2%)	39 (27.7%)	32 (27.1%)	0.923
diuretics, n (%)	161 (61.7%)	81 (57.4%)	79 (66.9%)	0.116
PDE5-inhibitors, n (%)	11 (4.2%)	4 (2.8%)	7 (5.9%)	0.234
dihydralazine, n (%)	76 (29.1%)	35 (24.8%)	41 (34.7%)	0.084
a1-blockers, n (%)	186 (71.3%)	89 (63.1%)	96 (81.4%)	<0.001
Ca2-channel-blockers, n (%)	179 (68.6%)	96 (68.1%)	83 (70.3%)	0.621
catecholamines, n (%)	102 (39.1%)	50 (35.5%)	51 (43.2%)	0.186

Abbreviations: AF = atrial fibrillation; BMI = body mass index; bpm = beats per minute; CLAS-ICH = causal classification system for intracerebral hemorrhage subtypes; DWM = deep white matter; EVD = external ventricular drain; GCS = Glasgow Coma Scale; HR = heart rate; HRV = heart rate variability; ICH = intracerebral hemorrhage; ICU = intensive care unit; INR = international normalized ratio; IVH = intraventricular hemorrhage; mRS = modified Rankin Scale; NIHSS = National Institutes of Health Stroke Scale; NSAID = non-steroidal anti-inflammatory drug; PCC = prothrombin complex concentrate; pmRS = modified Rankin Scale before the index event; PTT = partial thromboplastin time; PWM = periventricular white matter; SD = standard deviation.

**Table 2:** Absolute heart rate at admission and HRV indices in patients with ICH, based on good versus poor functional outcome (mRS 0–2 versus 3–6) at 90 days follow-up.

	All ICH Patients (n = 261)	ICH Patients with Good Outcome after 90 days (n = 106)	ICH Patients with Poor Outcome after 90 days (n = 155)	*p*-Value
**at admission**				
HR in bpm, mean ± SD	79.1 ± 16.5	77.9 ± 13.3	79.8 ± 15.9	0.360
**time interval 0–2 h**				
HR SV in ms, mean ± SD	8.6 ± 5.7	8.5 ± 6.4	8.7 ± 5.3	0.830
HR SD in bpm, mean ± SD	8.3 ± 4.5	8.2 ± 4.9	8.4 ± 4.3	0.655
HR CV in %, mean ± SD	0.1 ± 0.1	0.1 ± 0.1	0.1 ± 0.1	1.000
HR in bpm, mean ± SD	78.6 ± 13.3	77.3 ± 14.8	79.4 ± 12.1	0.222
**time interval 0–8 h**				
HR SV in ms, mean ± SD	8.8 ± 5.4	8.5 ± 5.9	8.9 ± 5.1	0.599
HR SD in bpm, mean ± SD	8.8 ± 4.3	8.7 ± 4.6	9.0 ± 4.2	0.594
HR CV in %, mean ± SD	0.1 ± 0.1	0.1 ± 0.1	0.1 ± 0.1	0.982
HR in bpm, mean ± SD	78.2 ± 13.1	76.8 ± 14.5	79.1 ± 12.0	0.172
**time interval 0–12 h**				
HR SV in ms, mean ± SD	9.4 ± 5.5	9.0 ± 5.3	9.6 ± 5.6	0.397
HR SD in bpm, mean ± SD	10.1 ± 4.6	9.6 ± 4.3	10.4 ± 4.8	0.185
HR CV in %, mean ± SD	0.1 ± 0.1	0.1 ± 0.1	0.1 ± 0.1	0.387
HR in bpm, mean ± SD	76.6 ± 12.3	75.3 ± 13.5	77.5 ± 11.3	0.161
**time interval 0–24 h**				
HR SV in ms, mean ± SD	9.9 ± 4.8	9.7 ± 4.6	10.0 ± 4.9	0.675
HR SD in bpm, mean ± SD	10.9 ± 4.1	10.3 ± 3.9	11.4 ± 4.2	0.037
HR CV in %, mean ± SD	0.1 ± 0.1	0.1 ± 0.0	0.2 ± 0.1	0.131
HR in bpm, mean ± SD	75.1 ± 12.0	73.5 ± 12.8	76.1 ± 11.3	0.090
**time interval 0–48 h**				
HR SV in ms, mean ± SD	9.9 ± 4.4	9.8 ± 4.2	9.9 ± 4.6	0.834
HR SD in bpm, mean ± SD	11.1 ± 3.8	10.4 ± 3.5	11.7 ± 3.9	0.008
HR CV in %, mean ± SD	0.2 ± 0.1	0.1 ± 0.0	0.2 ± 0.1	0.040
HR in bpm, mean ± SD	74.6 ± 11.7	73.0 ± 12.2	75.8 ± 11.2	0.063
**time interval 0–72 h**				
HR SV in ms, mean ± SD	9.9 ± 4.3	9.9 ± 4.1	9.9 ± 4.5	0.983
HR SD in bpm, mean ± SD	11.4 ± 3.9	10.6 ± 3.5	12.0 ± 4.0	0.004
HR CV in %, mean ± SD	0.2 ± 0.1	0.1 ± 0.1	0.2 ± 0.1	0.018
HR in bpm, mean ± SD	74.3 ± 11.2	72.8 ± 11.7	75.4 ± 10.7	0.064
**time interval 8–16 h**				
HR SV in ms, mean ± SD	8.9 ± 6.6	8.4 ± 5.0	9.2 ± 7.5	0.390
HR SD in bpm, mean ± SD	7.7 ± 5.1	7.2 ± 3.8	8.0 ± 5.8	0.212
HR CV in %, mean ± SD	0.1 ± 0.1	0.1 ± 0.1	0.1 ± 0.1	0.457
HR in bpm, mean ± SD	75.3 ± 13.7	73.6 ± 14.4	76.5 ± 13.2	0.100
**time interval 16–24 h**				
HR SV in ms, mean ± SD	9.1 ± 6.4	8.5 ± 5.6	9.5 ± 6.9	0.215
HR SD in bpm, mean ± SD	7.6 ± 4.6	7.0 ± 3.8	8.1 ± 5.1	0.059
HR CV in %, mean ± SD	0.1 ± 0.1	0.1 ± 0.1	0.1 ± 0.1	0.109
HR in bpm, mean ± SD	73.6 ± 13.1	72.9 ± 12.8	74.1 ± 13.2	0.491
**time interval 24–48 h**				
HR SV in ms, mean ± SD	9.9 ± 5.2	10.0 ± 5.0	9.8 ± 5.4	0.789
HR SD in bpm, mean ± SD	9.3 ± 3.8	9.0 ± 3.5	9.5 ± 4.0	0.335
HR CV in %, mean ± SD	0.1 ± 0.1	0.1 ± 0.1	0.1 ± 0.1	0.929
HR in bpm, mean ± SD	72.8 ± 13.3	70.7 ± 12.2	74.1 ± 13.2	0.053
**time interval 48–72 h**				
HR SV in ms, mean ± SD	9.4 ± 5.2	9.9 ± 5.9	9.1 ± 4.7	0.272
HR SD in bpm, mean ± SD	9.1 ± 4.0	8.8 ± 3.8	9.2 ± 4.1	0.503
HR CV in %, mean ± SD	0.1 ± 0.1	0.1 ± 0.1	0.1 ± 0.1	0.830
HR in bpm, mean ± SD	73.1 ± 12.4	70.7 ± 11.0	74.4 ± 13.0	0.034
**time interval 72–96 h**				
HR SV in ms, mean ± SD	9.3 ± 5.7	9.7 ± 6.7	9.1 ± 5.1	0.484
HR SD in bpm, mean ± SD	8.9 ± 4.7	8.8 ± 4.9	9.0 ± 4.7	0.742
HR CV in %, mean ± SD	0.1 ± 0.1	0.1 ± 0.1	0.1 ± 0.1	0.851
HR in/min, mean ± SD	73.0 ± 12.0	70.7 ± 10.2	74.3 ± 12.8	0.041

Abbreviations: bpm = beats per minute; CV = coefficient of variation: ICH = intracerebral hemorrhage; HR = heart rate; SD = standard deviation; SV = successive variability.

**Table 3:** Univariate analysis of different HRV indices on good outcome at follow-up.

	OR	CI down	CI up	*p*-Value
SV HRV 0–2 h	0.995	0.953	1.040	0.829
SD HRV 0–2 h	0.987	0.934	1.044	0.654
CV HRV 0–2 h	1.000	0.013	74.145	1.000
mean HR 0–2 h	0.988	0.970	1.007	0.222
SV HRV 0–8 h	0.987	0.942	1.035	0.598
SD HRV 0–8 h	0.984	0.929	1.043	0.593
CV HRV 0–8 h	0.948	0.010	86.162	0.982
mean HR 0–8 h	0.987	0.968	1.006	0.172
SV HRV 0–16 h	0.980	0.935	1.027	0.397
SD HRV 0–16 h	0.963	0.910	1.019	0.186
CV HRV 0–16 h	0.150	0.002	11.038	0.387
mean HR 0–16 h	0.985	0.965	1.006	0.162
SV HRV 0–24 h	0.989	0.939	1.042	0.674
SD HRV 0–24 h	0.934	0.876	0.997	0.039
CV HRV 0–24 h	0.027	0.000	2.973	0.133
mean HR 0–24 h	0.982	0.961	1.003	0.092
SV HRV 0–48 h	0.994	0.939	1.052	0.833
SD HRV 0–48 h	0.910	0.848	0.976	0.009
CV HRV 0–48 h	0.005	0.000	0.834	0.042
mean HR 0–48 h	0.979	0.958	1.001	0.065
SV HRV 0–72 h	0.999	0.944	1.058	0.983
SD HRV 0–72 h	0.903	0.841	0.969	0.005
CV HRV 0–72 h	0.002	0.000	0.383	0.020
mean HR 0–72 h	0.979	0.956	1.001	0.066
SV HRV 8–16 h	0.982	0.941	1.024	0.395
SD HRV 8–16 h	0.964	0.910	1.022	0.219
CV HRV 8–16 h	0.202	0.003	13.669	0.457
mean HR 8–16 h	0.984	0.965	1.003	0.102
SV HRV 16–24 h	0.973	0.932	1.016	0.218
SD HRV 16–24 h	0.944	0.888	1.003	0.063
CV HRV 16–24 h	0.027	0.000	2.378	0.114
mean HR 16–24 h	0.993	0.974	1.013	0.489
SV HRV 24–48 h	1.007	0.958	1.058	0.788
SD HRV 24–48 h	0.966	0.901	1.036	0.335
CV HRV 24–48 h	0.802	0.006	101.807	0.929
mean HR 24–48 h	0.980	0.960	1.000	0.055
SV HRV 48–72 h	1.029	0.977	1.085	0.281
SD HRV 48–72 h	0.976	0.910	1.047	0.502
CV HRV 48–72 h	1.685	0.015	195.221	0.830
mean HR 48–72 h	0.975	0.953	0.998	0.035
SV HRV 72–96 h	1.018	0.969	1.070	0.484
SD HRV 72–96 h	0.990	0.930	1.053	0.740
CV HRV 72–96 h	1.574	0.014	172.725	0.850
mean HR 72–96 h	0.974	0.949	0.999	0.043

Abbreviations: CI = confidence interval; CV = coefficient of variation; HR = heart rate; HRV: heart rate variability; OR = odds ratio; SD = standard deviation; SV = successive variability.

## Data Availability

The data presented in this study are available on request from the corresponding author due to the fact that the data set contains additional information that is currently under revision and further planned to be published in future publications.

## Supplementary Materials

The following supporting information can be downloaded at: https://www.mdpi.com/article/10.3390/biomedicines12081877/s1, Table S1: Heart rate at admission and heart rate variability indices in patients with ICH, based on AF. Table S2: Heart rate at admission and heart rate variability indices in patients with ICH, based on sex.

## Abbreviations

CI = confidence interval; OAC = oral anticoagulants.
