# Supplementary material for: Heart Rate Variability and Functional Outcomes of Patients with Spontaneous Intracerebral Hemorrhage
Source: Biomedicines. 2024 Aug 16;12(8):1877. doi: 10.3390/biomedicines12081877 (PMC11351286; doi:10.3390/biomedicines12081877)
Supplement: Supplementary file 1 [file biomedicines-12-01877-s001.zip › biomedicines-3146129-supplementary.pdf]

Supplemental table S1: Heart rate at admission and heart rate variability indices in patients with ICH, based on AF.

.	all ICH-patients (n=261).	ICH-patients with AF (n=67).	ICH Patients without AF (n=194).	p-value..
**at admission..**	.	.	.	.
HR in bpm, mean±SD.	79.1±16.5.	81.0±18.7.	78.35±15.6.	0.002..
**time interval 0-2h..**	.	.	.	.
HR SV in ms, mean±SD.	8.6±5.7.	9.6±6.7.	8.3±5.4.	0.921..
HR SD in bpm, mean±SD.	8.3±4.5.	8.5±5.2.	8.3±4.3.	0.080..
HR CV in %, mean±SD.	0.1±0.1.	0.1±0.1.	0.1±0.1.	0.087..
HR in bpm, mean±SD.	78.6±13.3.	80.4±15.4.	77.9±12.4.	0.116..
**time interval 0-8h..**	.	.	.	.
HR SV in ms, mean±SD.	8.8±5.4.	9.8±6.3.	8.4±5.0.	0.930..
HR SD in bpm, mean±SD.	8.8±4.3.	9.2±4.7.	8.8±4.2.	0.051..
HR CV in %, mean±SD.	0.1±0.1.	0.1±0.1.	0.1±0.1.	0.055..
HR in bpm, mean±SD.	78.2±13.1.	80.0±15.4.	77.5±12.1.	0.073..
**time interval 0-12h..**	.	.	.	.
HR SV in ms, mean±SD.	9.4±5.5.	10.2±5.8.	9.1±5.3.	0.965..
HR SD in bpm, mean±SD.	10.1±4.6.	10.6±4.7.	9.9±4.5.	0.038..
HR CV in %, mean±SD.	0.1±0.1.	0.1±0.1.	0.1±0.1.	0.041..
HR in bpm, mean±SD.	76.6±12.3.	78.2±14.5.	76.1±11.4.	0.012..
**time interval 0-24h..**	.	.	.	.
HR SV in ms, mean±SD.	9.9±4.8.	10.9±5.2.	9.5±4.6.	0.705..
HR SD in bpm, mean±SD.	10.9±4.1.	11.6±4.4.	10.7±4.0.	0.068..
HR CV in %, mean±SD.	0.1±0.1.	0.2±0.1.	0.1±0.1.	0.081..
HR in bpm, mean±SD.	75.1±12.0.	76.6±14.0.	74.5±11.2.	0.002..
**time interval 0-48h..**	.	.	.	.
HR SV in ms, mean±SD.	9.9±4.4.	10.7±4.7.	9.6±4.3.	0.222..
HR SD in bpm, mean±SD.	11.1±3.8.	11.8±3.9.	10.9±3.7.	0.154..
HR CV in %, mean±SD.	0.2±0.1.	0.2±0.0.	0.1±0.1.	0.179..
HR in bpm, mean±SD.	74.6±11.7.	75.9±13.5.	74.2±11.0.	<0.001..
**time interval 0-72h..**	.	.	.	.
HR SV in ms, mean±SD.	9.9±4.3.	10.7±4.5.	9.6±4.3.	0.193..
HR SD in bpm, mean±SD.	11.4±3.9.	12.3±4.4.	11.1±3.6.	0.115..
HR CV in %, mean±SD.	0.2±0.1.	0.2±0.0.	0.2±0.1.	0.140..
HR in bpm, mean±SD.	74.3±11.2.	76.0±13.1.	73.8±10.4.	<0.001..
**time interval 8-16h..**	.	.	.	.
HR SV in ms, mean±SD.	8.9±6.6.	9.5±5.2.	8.6±7.1.	0.553..
HR SD in bpm, mean±SD.	7.7±5.1.	8.4±4.1.	7.5±5.4.	0.679..
HR CV in %, mean±SD.	0.1±0.1.	0.1±0.1.	0.1±0.1.	0.690..
HR in bpm, mean±SD.	75.3±13.7.	77.0±14.8.	74.7±13.3.	0.020..
**time interval 16-24h..**	.	.	.	.
HR SV in ms, mean±SD.	9.1±6.4.	9.7±6.7.	8.9±6.3.	0.491..

HR SD in bpm, mean±SD.	7.6±4.6.	8.2±5.1.	7.4±4.4.	0.500..
HR CV in %, mean±SD.	0.1±0.1.	0.1±0.1.	0.1±0.1.	0.496..
HR in bpm, mean±SD.	73.6±13.1.	74.3±14.8.	73.3±12.3.	0.030..
**time interval 24-48h..**	**.**	.	.	.
HR SV in ms, mean±SD.	9.9±5.2.	10.7±6.1.	9.6±4.8.	0.063..
HR SD in bpm, mean±SD.	9.3±3.8.	9.9±4.5.	9.0±3.5.	0.846..
HR CV in %, mean±SD.	0.1±0.1.	0.1±0.1.	0.1±0.1.	0.779..
HR in bpm, mean±SD.	72.8±13.3.	73.9±14.6.	72.4±12.8.	<0.001..
**time interval 48-72h..**	**.**	.	.	.
HR SV in ms, mean±SD.	9.4±5.2.	8.8±4.0.	9.7±5.6.	0.020..
HR SD in bpm, mean±SD.	9.1±4.0.	8.4±3.8.	9.3±4.0.	0.229..
HR CV in %, mean±SD.	0.1±0.1.	0.1±0.0.	0.1±0.1.	0.217..
HR in bpm, mean±SD.	73.1±12.4.	73.6±13.2.	72.8±12.1.	<0.001..
**time interval 72-96h..**	**.**	.	.	.
HR SV in ms, mean±SD.	9.3±5.7.	9.4±4.8.	9.3±6.0.	0.375..
HR SD in bpm, mean±SD.	8.9±4.7.	9.2±5.3.	8.8±4.5.	0.031..
HR CV in %, mean±SD.	0.1±0.1.	0.1±0.1.	0.1±0.1.	0.034..
HR in bpm, mean±SD.	73.0±12.0.	75.5±14.9.	72.0±10.6.	0.002..

Abbreviations: AF = atrial fibrillation bpm = beats per minute; CV = coefficient of variation: ICH = intracerebral hemorrhage; HR = heart rate; SD = standard deviation; SV = successive variability.

Supplemental table S2: Heart rate at admission and heart rate variability indices in patients with ICH, based on sex.

.	all ICH-patients (n=261).	female ICH-patients (n=127, 48.7%).	male ICH Patients (n=134, 51.3%).	p-value..
**at admission..**	.	.	.	.
HR in bpm, mean±SD.	79.1±16.5.	79.4±16.7.	78.8±16.4.	0.360..
**time interval 0-2h..**	.	.	.	.
HR SV in ms, mean±SD.	8.6±5.7.	8.6±5.7.	8.6±5.8.	0.830..
HR SD in bpm, mean±SD.	8.3±4.5.	8.3±4.3.	8.3±4.7.	0.655..
HR CV in %, mean±SD.	0.1±0.1.	0.1±0.1.	0.1±0.1.	1.000..
HR in bpm, mean±SD.	78.6±13.3.	78.4±12.2.	78.7±14.2.	0.222..
**time interval 0-8h..**	.	.	.	.
HR SV in ms, mean±SD.	8.8±5.4.	8.6±5.3.	8.9±5.5.	0.599..
HR SD in bpm, mean±SD.	8.8±4.3.	8.8±4.2.	9.0±4.5.	0.594..
HR CV in %, mean±SD.	0.1±0.1.	0.1±0.1.	0.1±0.1.	0.982..
HR in bpm, mean±SD.	78.2±13.1.	77.9±11.8.	78.4±14.2.	0.172..
**time interval 0-12h..**	.	.	.	.
HR SV in ms, mean±SD.	9.4±5.5.	9.5±5.8.	9.3±5.2.	0.397..
HR SD in bpm, mean±SD.	10.1±4.6.	9.9±4.8.	10.3±4.4.	0.185..
HR CV in %, mean±SD.	0.1±0.1.	0.1±0.1.	0.1±0.1.	0.387..
HR in bpm, mean±SD.	76.6±12.3.	76.6±10.8.	76.6±13.5.	0.161..
**time interval 0-24h..**	.	.	.	.
HR SV in ms, mean±SD.	9.9±4.8.	10.0±5.1.	9.8±4.6.	0.675..
HR SD in bpm, mean±SD.	10.9±4.1.	10.7±4.1.	11.1±4.0.	0.037..
HR CV in %, mean±SD.	0.1±0.1.	0.1±0.1.	0.1±0.1.	0.131..
HR in bpm, mean±SD.	75.1±12.0.	75.1±10.4.	75.0±13.4.	0.090..
**time interval 0-48h..**	.	.	.	.
HR SV in ms, mean±SD.	9.9±4.4.	10.1±4.6.	9.6±4.2.	0.834..
HR SD in bpm, mean±SD.	11.1±3.8.	11.1±3.9.	11.2±3.7.	0.008..
HR CV in %, mean±SD.	0.2±0.1.	0.2±0.1.	0.2±0.0.	0.040..
HR in bpm, mean±SD.	74.6±11.7.	74.7±10.1.	74.6±13.0.	0.063..
**time interval 0-72h..**	.	.	.	.
HR SV in ms, mean±SD.	9.9±4.3.	10.1±4.4.	9.8±4.3.	0.983..
HR SD in bpm, mean±SD.	11.4±3.9.	11.3±3.8.	11.5±3.9.	0.004..
HR CV in %, mean±SD.	0.2±0.1.	0.2±0.1.	0.2±0.1.	0.018..
HR in bpm, mean±SD.	74.3±11.2.	74.3±9.7.	74.3±12.5.	0.064..
**time interval 8-16h..**	.	.	.	.
HR SV in ms, mean±SD.	8.9±6.6.	9.0±7.7.	8.8±5.6.	0.390..
HR SD in bpm, mean±SD.	7.7±5.1.	7.9±5.8.	7.6±4.3.	0.212..
HR CV in %, mean±SD.	0.1±0.1.	0.1±0.1.	0.1±0.1.	0.457..
HR in bpm, mean±SD.	75.3±13.7.	75.0±12.5.	75.6±14.8.	0.100..
**time interval 16-24h..**	.	.	.	.
HR SV in ms, mean±SD.	9.1±6.4.	9.1±6.0.	9.2±6.7.	0.215..
HR SD in bpm, mean±SD.	7.6±4.6.	7.7±4.6.	7.6±4.7.	0.059..
HR CV in %, mean±SD.	0.1±0.1.	0.1±0.1.	0.1±0.1.	0.109..

HR in bpm, mean±SD.	73.6±13.1.	74.3±11.3.	72.9±14.4.	0.491..
**time interval 24-48h..**	**.**	.	.	.
HR SV in ms, mean±SD.	9.9±5.2.	10.1±5.4.	9.7±5.1.	0.789..
HR SD in bpm, mean±SD.	9.3±3.8.	9.3±3.8.	9.3±3.9.	0.335..
HR CV in %, mean±SD.	0.1±0.1.	0.1±0.1.	0.1±0.1.	0.929..
HR in bpm, mean±SD.	72.8±13.3.	73.1±11.8.	72.5±14.6.	0.053..
**time interval 48-72h..**	**.**	.	.	.
HR SV in ms, mean±SD.	9.4±5.2.	9.9±5.0.	9.1±5.4.	0.272..
HR SD in bpm, mean±SD.	9.1±4.0.	9.3±4.3.	8.8±3.7.	0.503..
HR CV in %, mean±SD.	0.1±0.1.	0.1±0.1.	0.1±0.1.	0.830..
HR in bpm, mean±SD.	73.1±12.4.	73.5±11.5.	72.7±13.2.	0.034..
**time interval 72-96h..**	**.**	.	.	.
HR SV in ms, mean±SD.	9.3±5.7.	9.4±4.8.	9.2±6.4.	0.484..
HR SD in bpm, mean±SD.	8.9±4.7.	9.2±4.7.	8.7±4.8.	0.742..
HR CV in %, mean±SD.	0.1±0.1.	0.1±0.1.	0.1±0.1.	0.851..
HR in bpm, mean±SD.	73.0±12.0.	72.8±11.0.	73.2±12.9.	0.041..

Abbreviations: Bpm = beats per minute; CV = coefficient of variation: ICH = intracerebral hemorrhage; HR = heart rate; SD = standard deviation; SV = successive variability.
